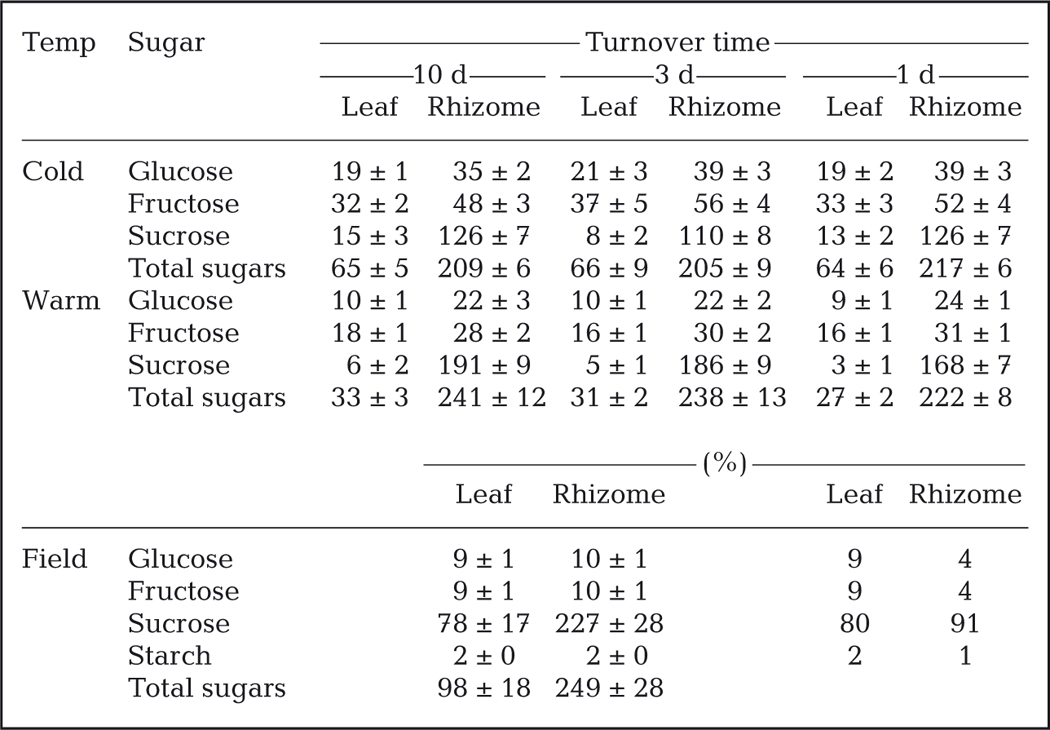# Carbon limitation in response to nutrient loading in an eelgrass mesocosm: influence of water residence time

**DOI:** 10.3354/meps14061

**Published:** 2022-05-12

**Authors:** James E. Kaldy, Cheryl A. Brown, Stephen R. Pacella

**Affiliations:** Pacific Ecological Systems Division, US EPA, 2111 SE Marine Science Center Dr., Newport, OR 97365, USA

**Keywords:** Eelgrass, *Zostera marina*, Seagrass, Nutrient loading, Eutrophication, Carbon limitation, Basification

## Abstract

Altered primary productivity associated with eutrophication impacts not only ecosystem structure but also the biogeochemical cycling of oxygen and carbon. We conducted laboratory experiments to empirically determine how residence time (1, 3, 10 d) influences eutrophication responses in a simplified Pacific Northwest *Zostera marina*–green macroalgal community. We expected long-residence time (RT) systems to exhibit eutrophication impairments. Instead, we observed an accumulation of nutrients at all RTs and a shift in the dissolved inorganic carbon speciation away from CO_2 (aq)_ with unexpected consequences for eel grass plant condition, including shoot mortality. Most metrics responded more strongly to temperature treatments than to RT treatments. No dramatic shifts in the relative abundance of *Z. marina* and green macro algae were detected. *Z. marina* shoot density proliferated in cool temperatures (12°C) with a modest decline at 20°C. Eelgrass loss was associated with high total scale pH (pH_T_) and CO_2 (aq)_ concentrations of <10 μmol kg^−1^ CO_2 (aq)_, but not with high nutrients. *Z. marina* δ^13^C values support the hypo thesis that carbon availability was greater at short RT. Further, very low leaf sugar concentrations are consistent with extreme photosynthetic CO_2 (aq)_ limitation. We suggest that the effects of extremely low environ mental car bon concentrations (CO_2 (aq)_) and increased respiration at warm temperatures (20°C) and other physiological processes can lead to internal carbon limitation and shoot mortality. Eutrophication responses to nutrient loading are more nuanced than just light limitation of eelgrass and require additional research on the interaction of the biogeochemical environment and plant physiology to better understand estuarine ecosystem disruption.

## Graphical Abstract

Photosynthetic production stimulated by nutrients and warming can lead to extreme CO_2_ limitation and eelgrass death.

Graphic: J. E. Kaldy with symbols from ian.umces.edu/media-library/symbols

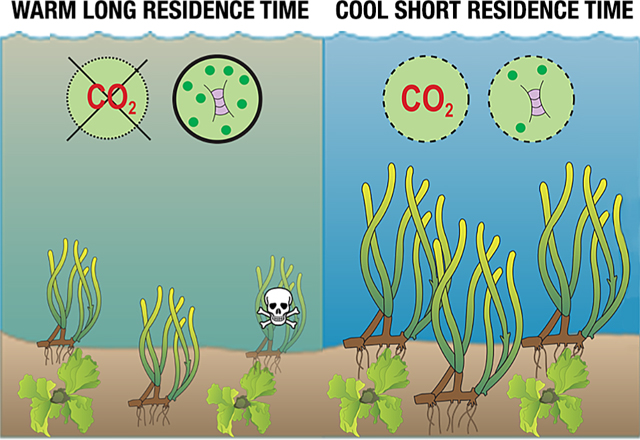

## 1. INTRODUCTION

Seagrasses are flowering plants that colonize shallow marine and estuarine environments (den Hartog & Kuo 2006) and are widely viewed as important contributors of ecosystem services, providing shoreline protection, fisheries production, carbon sequestration, and nutrient removal (Orth et al. 2006, Barbier et al. 2011). Seagrasses are often regarded as sentinel or indicator species because they are sensitive to anthropogenic perturbations and local water quality conditions. Alterations in the delivery and biogeochemical cycling of nutrients and carbon (e.g. eutro phication) can result in structural and functional changes in estuarine ecosystems including the de gradation and loss of seagrass communities. It is commonly accepted that increased nutrient loading alters primary production and results in the loss of seagrass through light limitation and replacement with either macroalgae or phytoplankton.

Eutrophication can be defined as ‘an increase in the rate of supply of organic matter to an ecosystem’ (Nixon 1995, p. 199), which emphasizes that this is a process and explicitly differentiates the process from the causes (e.g. nutrient enrichment, reduced grazing, longer residence time, etc.) and consequences (e.g. hypoxia, fish kills, turbidity, etc., Nixon 2009). Anthropogenic nutrient loading is considered the primary driver of estuarine eutrophication. Ecosystem susceptibility to eutrophication driven by anthropogenic nutrient loading is strongly related to water residence time within the system, such that at longer residence times, eutrophication responses are expected to be more severe (Valiela et al. 1997, Krause-Jensen et al. 2008, Scavia & Liu 2009, Nidzieko 2018). Expected eutrophication responses due to accumulation of organic matter include large diurnal variations in the oxygen and carbon cycles, floating algal mats, phytoplankton blooms, and community shifts. However, the spatial and temporal dynamics of carbonate chemistry patterns in estuaries are more complex than in the open ocean (Cai et al. 2011, Waldbusser & Salisbury 2014), and the response of carbonate speciation is likely to be different from the open ocean (Feely et al. 2010). Changes in the relative abundance or availability of inorganic carbon ions in seawater may exacerbate photosynthetic carbon limitation driving eelgrass into negative carbon balance.

Eelgrass *Zostera marina* carbon budgets are the balance between carbon fixation (photosynthesis), growth, storage, and losses such as respiration and exudation. Photosynthetic rates can be limited by light, carbon supply, and to an extent by temperature (Zimmerman 2017). Under low light levels, plants may be unable to maintain a positive carbon balance and can utilize stored reserves if available but will eventually die if deprived of light for extended periods (Herzka & Dunton 1998, Cabello-Pasini et al. 2002, Eriander 2017). Similarly, the availability of CO_2 (aq)_ can limit seagrass photosynthetic rates. *Z. marina* is generally considered carbon limited, preferentially utilizes CO_2 (aq)_, and has a limited capacity to utilize bicarbonate (HCO_3_^−^) via external carbonic anhydrase (Beer & Koch 1996, Invers et al. 2001, Palacios & Zimmerman 2007). Although *Z. marina* does not appear to have a carbon-concentrating mechanism (CCM) in the cytoplasm or chloroplasts (Larkum et al. 2017), HCO_3_^−^ use can account for about 50% of light-saturated carbon fixation from CO_2_ (Invers et al. 2001). Other physiological constraints on the plant carbon balance include the influence of increased photorespiration (Buchanan et al. 2000, Touchette & Burkholder 2000a) and warming, which increases the rate of respiration faster than the rate of photosynthesis (Lee et al. 2007).

Despite the recognized importance of residence time as a control on the expression of eutrophication, almost no empirical work has been conducted to evaluate these relationships. Pioneering mesocosm studies on seagrass community response to nutrient loading were conducted at long residence times (10–20 d) and elevated temperatures simulating quiescent embayments along the US Atlantic coast (Burkholder et al. 1992, Short et al. 1995, Taylor et al. 1995). Estuaries along the West Coast of North America are distinct from other regions; they tend to be small, have short residence times, and are mesotidal with large nutrient inputs associated with cold, recently upwelled water (Hickey & Banas 2003) and N-fixation by trees in the watershed (Lee & Brown 2009). Recent experiments concluded that despite massive nutrient loads (up to 50 mmol NO_3_ d^−1^ to 325 l tanks), eutrophication responses for seagrass communities were subtle or not detectable at high water turnover rates (Kaldy et al. 2017). A better understanding of how water residence time controls nutrient concentrations and ultimately eutrophication response is critical for estuarine nutrient management.

Burkholder et al. (1992) described eelgrass shoot death in response to low level nitrate enrichment. Later work found that *Z. marina* did not down-regulate nitrate reductase and as a result did not control nitrate assimilation which requires carbon skeletons potentially leading to negative internal carbon balance (Touchette & Burkholder 2007). Other studies invoked similar mechanisms to explain ‘ammonium toxicity’ in several seagrass species (van Katwijk et al. 1997, Brun et al. 2002, Christianen et al. 2011). However, Kaldy (2014) reported that *Z. marina* did not exhibit mortality at extreme NO_3_^−^ or NH_4_^+^ concentrations when grown with saturating irradiance, cool water temperatures, and aeration (CO_2_ replete). Other work showed that *Z. marina* mortality response to reduced nitrogen (e.g. NH_4_) was highly dependent on pH, which is a proxy for carbonate chemistry conditions (van der Heide et al. 2008, Christianen et al. 2011). Researchers rarely quantify the complete carbonate chemistry of seagrass experimental systems and often rely on pH as a proxy that alone does not adequately describe the carbon ion speciation and availability. Consequently, we believe that shoot mortality could be related to carbon limitation and may not necessarily be a direct ‘toxicity’ effect.

Estuarine communities respond to a wide variety of natural and anthropogenic stressors, and these responses are often constrained by physical conditions such as temperature, light, and circulation patterns. Population-level responses are an integration of external forcing factors as well as physiological tolerances. We designed these experiments to empirically evaluate how a simplified *Z. marina*, macroalgae, phytoplankton system responds to and alters carbon biogeochemistry under high nutrient loads and variable residence times. Experimental conditions were chosen to mimic characteristics of Pacific Northwest open coast estuaries with respect to light and temperature, while a range of residence times or turnover rates (how quickly water is replaced in the system) were chosen based on previous work. We hypothesized that eutrophication expression (e.g. algal accumulation, plankton blooms, dissolved oxygen (DO), carbonate chemistry, community shifts, etc.) would be related to nutrient concentration and water residence time within the mesocosms. Specifically, we expected long-residence time systems to exhibit the most severe alterations of biogeochemistry as well as eutrophication impairments.

## 2. MATERIALS AND METHODS

### 2.1. Mesocosm conditions

**Fig. 1.**
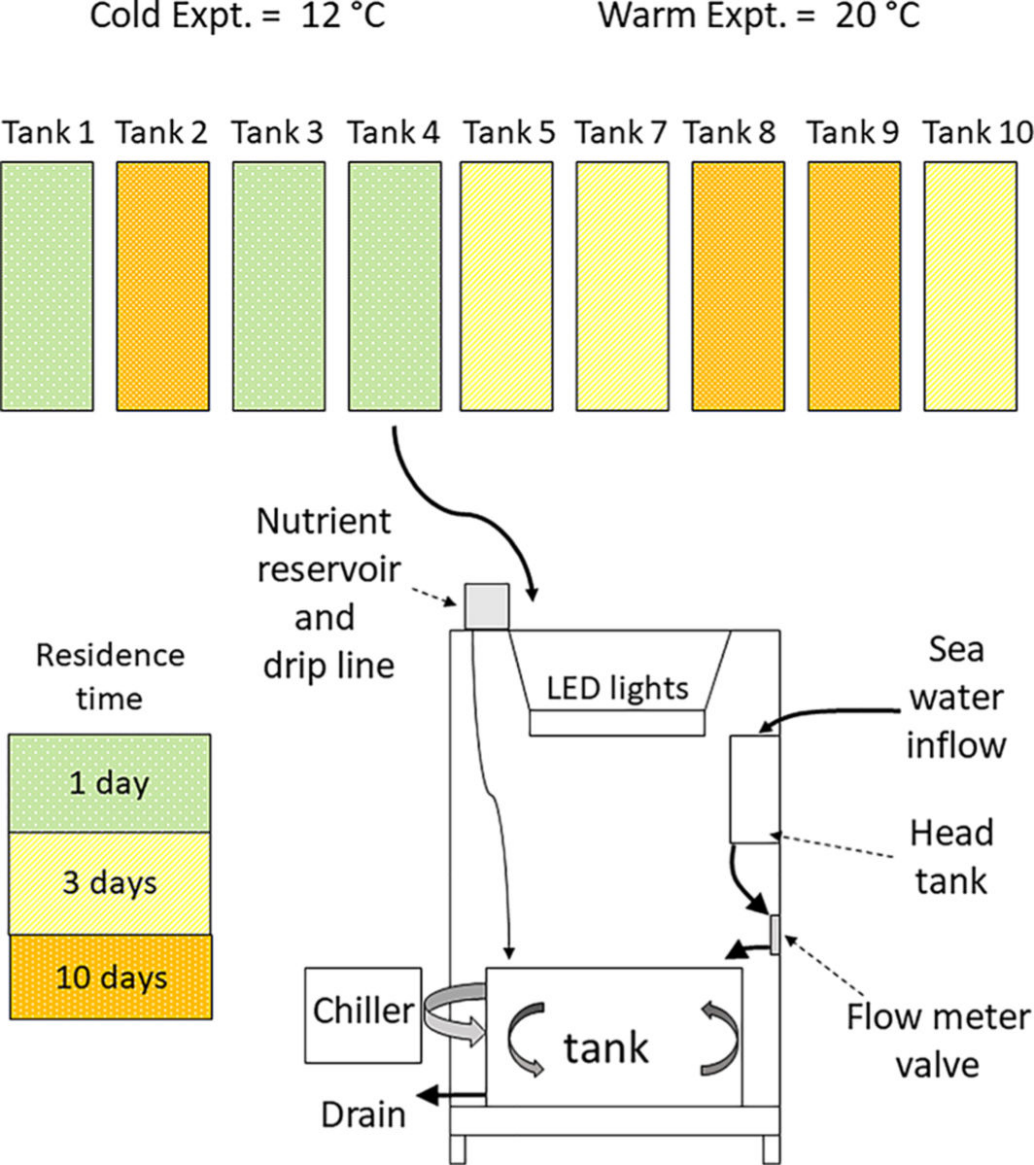
Experimental design. The cold experiment was conducted at 12°C and the warm experiment at 20°C. Residence time treatments are designated by shading. Inset panel is a schematic of each replicate mesocosm system. Note: Tank 6 was randomly excluded from these experiments

The experimental facility (described previously by Kaldy 2014 and Kaldy et al. 2017) consisted of 9 independent replicate cabinets (Fig. 1), each of which contained 2 banks of LED grow lights (180 W Advance Spectrum Max; AIBC International) suspended over a white polycarbonate tank (60 cm wide × 60 cm deep × 90 cm long; ~325 l). Each LED panel consisted of 119 LEDs in a 6:1:1:1 ratio of red (660 nm λ), blue (465 nm λ), orange (620 nm λ), and white (6000 K). Photosynthetically available scalar irradiance (400–700 nm λ) measured at the middle of the tank water column with a spherical quantum sensor (LI-193SA) and LI-1400 data logger (LI-COR) was >200 μmols photons m^−2^ s^−1^, which is above light saturation for *Zostera marina* and *Z. japonica* (Shafer & Kaldy 2014). Tanks were maintained on a 12:12 h light:dark cycle with a daily light-saturated photosynthesis (H_sat_) period of 12 h.

Each replicate mesocosm cabinet contained a polycarbonate tank, and the ambient seawater delivery rate was adjusted to achieve the target water residence times (described in Section 2.4). The cabinets had separate seawater head tanks that eliminated pressure changes, and the water delivery rate to each cabinet was controlled using adjustable acrylic flowmeters (Models FL2050 and FL2045, Omega). Inflow was checked daily using a graduated cylinder and adjusted as needed.

Nutrients were delivered to the tanks in 2 ways: from the ambient seawater and from the manipulated treatment load simulating ‘anthropogenic’ inputs. The Hatfield Marine Science Center seawater system pumps water from near the mouth of Yaquina Bay, OR, USA, and is collected daily during high tide. Seawater from this system is referred to as ‘source water’ throughout this paper. Ambient nutrient loading was a function of the source water delivery rate and the nutrient concentrations which varied as a result of upwelling/relaxation events on the shelf adjacent to Yaquina Bay. Because of inherent differences in nutrient load associated with altered residence time (nutrient load = nutrient concentration × daily input volume) the manipulated nutrient load had to exceed these potential confounding ambient loads. We chose to simulate nutrient loading as the maximum realistic load based on conditions in Yaquina Bay, where the plants were collected. Consequently, all mesocosm tanks received the same simulated anthropogenic nutrient load calculated ass uming 200% turnover d^−1^ (~650 l d^−1^) and a maximum ambient NO_3_ concentration of 30 μmols l^−1^ observed in Yaquina Bay (Brown & Ozretich 2009). To prevent phosphorus limitation, PO_4_ was added as KH_2_PO_4_ according to the Redfield ratio. This simulated anthropogenic nutrient load was ~20 mmol N d^−1^ and 1.2 mmol P d^−1^ delivered daily to each tank in a 4 l aliquot via a slow drip system throughout the course of the day (~20 h). Nutrient stock solution was mixed in 16 l batches consisting of 8.0 g KNO_3_ (MP Biomedicals) with a δ^15^N = 48.14 ± 0.12‰ and 0.7 g KH_2_PO_4_ (Fisher Scientific) dissolved in 16 l of milli-Q water (18 Ω).

Mixing and temperature control in each mesocosm was provided by submersible pumps (EcoPlus 633; Sunlight Supply) plumbed through an aquarium chiller (1/4 hp EcoPlus Chiller) with return flow spread diffusely along the bottom of the tank. Chillers were used to maintain water temperature below ambient room temperature for the cold experiment, while 300 W aquarium heaters were used to raise water temperature for the warm experiment. Additionally, each tank contained a wall-mounted wave maker (Koralia 1600; Hydor) to maintain mixing within the tank.

### 2.2. Phytoplankton and surface microalgae

To minimize variability between replicate mesocosms, we seeded the mesocosms with a cultured phytoplankton community. Briefly, we concentrated phytoplankton from the seawater system using a 10 μm mesh net. A 2 ml subsample was introduced to 1 l of pasteurized filtered seawater amended with 20 ml of F/2 media (Guillards F/2 marine water enrichment solution; Sigma Aldrich) with air mixing, 12:12 h light:dark cycle from 2 LED light panels at 12°C. After about 2 wk, the 1 l cultures were transferred to 2 carboys (20 l each) with 500 g Red Sea Salt® dissolved in 20 l milli-Q water (final salinity 25) amended with ~480 ml of F/2 media. Cultures were grown for another 10 to 14 d and then distributed to the tanks. During July, 1 of the 20 l phytoplankton culture carboys broke; as a result, 2 l of phytoplankton culture were added to each mesocosm. During the August experiment, 4 l of phytoplankton were added to each mesocosm. Microscope observation of subsamples revealed that pennate diatoms were the dominant phytoplankton in culture; a few flagellated cells were also observed. This was likely a ‘weedy’ culture of species selected by incubation conditions and is probably not representative of the natural field phytoplankton community. However, the culture provided an inoculum that could be expected to bloom under high nutrient loading conditions.

### 2.3. Macrophyte collection and initial conditions

For both experiments, fresh plant material was collected from Yaquina Bay during July and August 2016. During low tide, we collected ~200 shoots of *Z. marina* and a small bucket of drift green macroalgae (*Ulva* spp.) about 5 km from the estuary mouth. Plants were returned to the lab and held in flowing seawater tables overnight before being cleaned, processed, and distributed to mesocosms within 24 h. *Z. marina* plants were trimmed to consistent dimensions; old leaves and existing lateral or daughter shoots were removed, and the 3–4 youngest leaves were trimmed to 50 cm length measured from emerging rootlets and 5 rhizome internodes (Kaldy 2014). Epiphytes were removed by wiping both sides of the sheath and each leaf with a wet sponge. Two shoots of *Z. marina* were then transplanted into each of 90 plastic nursery pots (13 cm diameter × 11 cm deep), which were filled with estuarine sand. Each mesocosm tank contained 10 nursery pots with 20 *Z. marina* shoots. Green macroalgae (GMA) were rinsed with seawater over a 5 mm mesh to remove sediment and debris and picked through to remove invertebrates. Clean GMA were then spun in a salad spinner to remove water, and 50 g (wet weight) were added to each mesocosm tank.

### 2.4. Experimental design

We used a randomized factorial design (3 mesocosm replicates per treatment) to evaluate the effects of 3 water residence times (RTs: 1, 3, and 10 d) on expression of eutrophication in 9 mesocosms simulating estuarine macrophyte communities. To better understand how temperature influences eutrophication expression, 2 separate experiments (12°C: cold; 20°C: warm) were conducted. The cold experiment was conducted between 5 and 26 July 2016, while the warm experiment ran between 23 August and 16 September 2016. Temperature treatments were maintained within ±2°C using aquarium heaters or chillers as described above. All tanks received ambient seawater (sand filtered, ~70 μm) as supplied by the Hatfield Marine Science Center seawater system. Source water delivery rates, controlled by flow-meter valves, resulted in water RTs of 1 d (100% d^−1^ exchange, 225 ml min^−1^); 3 d (33% d^−1^ exchange; 75 ml min^−1^), and 10 d (10% d^−1^ exchange; 22 ml min^−1^). Each RT treatment was maintained in triplicate at a single temperature (12 or 20°C), and each experiment was run for 21 d.

### 2.5. Response metrics

Water was sampled daily from each tank and the source water and analyzed for dissolved inorganic nutrients (NO_2_^−^ + NO_3_^−^, NH_4_^+^, and PO_4_^−3^) and chlorophyll *a* (chl *a*) concentration. Throughout this manuscript, NO_3_ denotes NO_2_^−^ + NO_3_^−^, since NO_2_^−^ makes up only a small fraction of the total. Water aliquots (15 ml) were filtered (0.45 μm); filtrate was stored frozen at −20°C and subsequently analyzed using Lachat flow injection instrumentation at the Marine Science Institute Analytical Laboratory UC-Santa Barbara. Water column chl *a* was measured using the *in vivo* chl *a* module with a Trilogy fluorometer (Turner Designs) in relative fluorescence units (RFUs). We used a regression relationship between RFUs and extracted chl *a* (μg l^−1^) from the same sample to convert RFUs to water column chl *a* (*y* = 0.0021*x* – 0.215; r^2^ = 0.858; n = 93; Fig. S1 in the Supplement at www.int-res.com/articles/suppl/m689p001_supp.pdf). Subsamples used for developing this relationship were filtered through GF/F filters; the filters were placed in 15 ml centrifuge tubes and frozen until analyzed. Filters were cold extracted with 90% acetone and analyzed using a calibrated Trilogy fluorometer using the non-acidification module. The fluorometer is calibrated annually and was calibrated on 17 May 2016 using pure chl *a* extract (Turner Designs 10–850). Fluorometer calibration was checked before each use with a solid secondary standard (Turner Designs 8000–952) to verify performance following standard protocols. Water column total scale pH (pH_T_) was monitored continuously using Honeywell Durafet III pH electrodes interfaced to a UDA 2182 analyzer. Reported pH_T_ values were recorded from the UDA 2182 boxes during mid-afternoon and averaged across the replicate mesocosms (n = 3). Electrodes were calibrated prior to each experiment with temperature controlled (25°C) CO_2_ certified reference material (CRM; Batch 132) from UC-San Diego, and performance was verified at the end of each experiment using CRM. Total pH of the CRM was calculated using reported dissolved inorganic carbon (DIC), alkalinity, salinity, and measured temperature of CRM at the time of calibration using CO_2_SYS and constants described below. DO values were recorded from calibrated YSI 6600 sonde measurements made in the morning in the dark and in the late afternoon after about 6 h of light. Twice during each experiment, grab samples for carbonate chemistry were collected. Brown glass bottles (355 ml) were overflowed with at least 3 volumes of water siphoned out of each mesocosm tank, fixed with 30 μl HgCl_2_ and capped with a gas-tight lid. Analysis for *p*CO_2 (s.w.)_ and TCO_2_ was conducted using non-dispersive infra-red absorption (Hales et al. 2004, Bandstra et al. 2006) in the laboratory of Burke Hales at Oregon State University. The full carbonate system was calculated using CO_2_SYS with K_1_ and K_2_ constants of Millero (2010) and mineral solubility constants from Mucci (1983).

We measured biological response metrics for the seagrasses and macroalgae; final biomass, growth rates, non-structural carbohydrate content, C:N content, and δ^13^C and δ^15^N of algal thallus and eelgrass new leaf tissue. *Z. marina* leaf growth rates were measured as described by Kaldy (2014), while GMA growth rate was calculated as the change in biomass (g dry weight, DW) divided by the length of the experiment in days. Stable isotopes and tissue carbon and nitrogen content were measured by the Integrated Stable Isotope Research Facility at the Pacific Ecological Systems Division, US EPA, Corvallis, OR. For *Z. marina*, we also recorded the number of new lateral shoots formed during the experiment, but these were not included in growth estimates. Shoots were determined to be dead if the meristem was mushy or if they dissociated from the meristem. Likewise, we assessed the occurrence of wasting disease following Burdick et al. (1993).

*Z. marina* leaf and rhizome carbohydrate content was measured using HPLC methods on composite samples (n = 3) consisting of 3–5 individual shoots or rhizomes from an individual tank. Leaf and rhizome tissues were dried at 70°C and powdered using a mortar and pestle. Subsamples (100 mg) were ex tracted in triplicate using hot water (80°C, Sørensen et al. 2018); supernatant was pooled and then analyzed using HPLC to quantify individual sugars (sucrose, glucose, and fructose) at the Linus Pauling Institute, Oregon State University. Samples were se parated isocratically using 550 mM NaOH on an MA-1 column (4 mm × 250 mm) at 0.4 ml min^−1^ using a Dionex HPLC. Sugars were detected with a pulsed amperometric detector with quad potential and a disposable Au electrode. Mixed standards of glucose, fructose, and sucrose were prepared using commercially available compounds (Sigma-Aldrich). Standards were injected at 5 concentrations throughout the analysis to verify there were no changes in retention time or area counts over time. Starch content was not measured.

At the end of the experiment, we quantified the surface microalgae (SMA) as mg chl *a* cm^−2^ by scraping a 4.9 cm^2^ area of random locations on the tank wall (n = 5 per tank) and tank floor (n = 5), transferring the matrix to a GFF filter that was stored frozen until analysis. SMA chl *a* was analyzed as outlined by Janousek & Folger (2012) using a Trilogy fluorometer with the non-acidification module. Estimates of chl *a* cm^−2^ were then scaled to the surface area of the walls (17 500 cm^2^) and bottom (4700 cm^2^), respectively, and summed to provide an estimate of total SMA biomass as mg chl *a* tank^−1^. Seagrass epiphytes were not quantified.

### 2.6. Statistical analyses

Seagrass and algal biological response variables were analyzed using 2-way ANOVA with temperature and RT as the dependent variables. Assumptions of normality and homogeneity of variance were evaluated. In some cases, multiple non-parametric Kruskal-Wallis ANOVAs were used to evaluate main effects (temperature, RT); in all cases, results were consistent with parametric ANOVAs. Consequently, only parametric results are presented. When a main effect was significant, Tukey’s post hoc test was used to determine where treatment differences occurred. Results were assessed at α = 0.05. Statistical analysis was carried out using Origin Pro 2019 (OriginLab). All data presented as mean ± SE.

## 3. RESULTS

### 3.1. Biogeochemistry

**Fig. 2.**
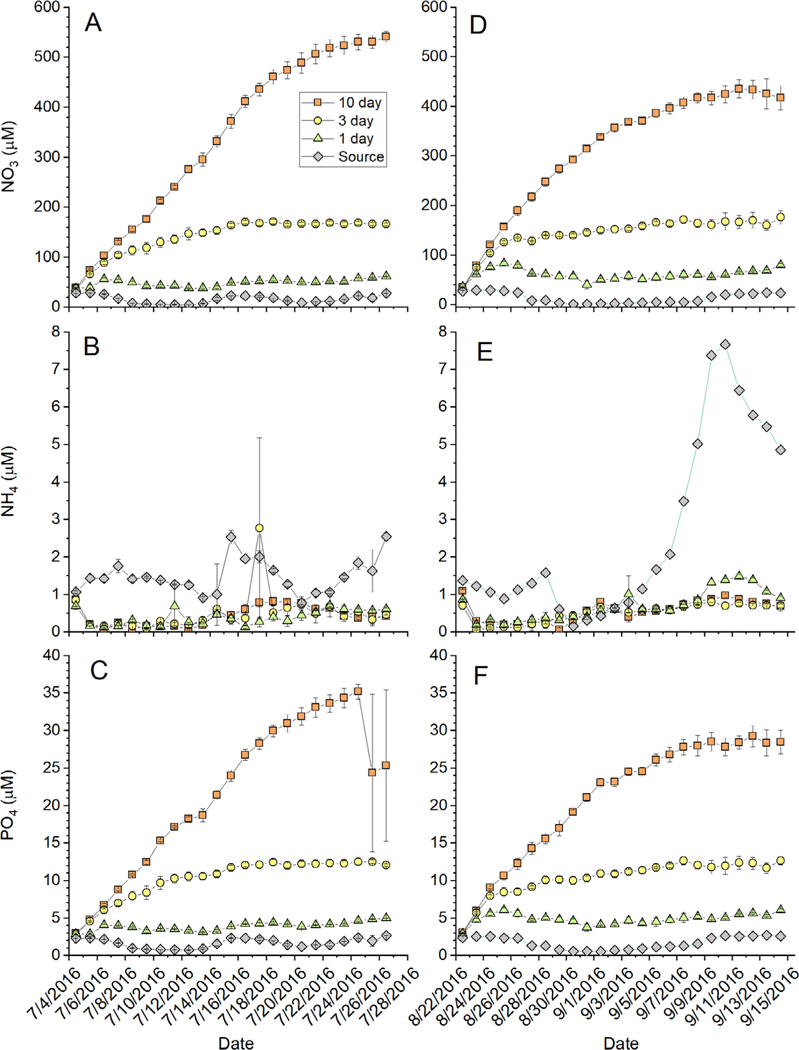
Time series (mean ± SE; n = 3) of water column NO_3_, NH_4_, and PO_4_ concentrations (μM) from the (A–C) cold and (D–F) warm experiments. Values are presented for each residence time treatment as well as the source water coming into the mesocosms. In some cases, the error bars are smaller than the symbol. Dates are given as mo/d/yr

Nominal nutrient loads were quite high as designed and similar between the cold and warm experiments (Table 1). Variability in inputs between experiments was related to changes in the upwelling/relaxation regime of the near shore source water. Highest daily loads were in the fast turnover tanks. Although the daily loading rates were quite high, the nutrient concentrations within the mesocosm tanks were dependent on RT treatment (Fig. 2). Source water nutrient concentrations were variable and ranged between 1 and 33 μM for NO_3_, 0.5 and 2.8 μM for PO_4_, and 0.2 and 7.7 μM for NH_4_ (Fig. 2). All RT treatments exhibited increased NO_3_ and PO_4_ concentrations relative to the source water, and the longer RT treatments (3 and 10 d RT) accumulated excessive nutrients. Nitrate concentrations in the 10 d RT treatment were around 400 to 500 μM by the end of the experiments (Fig. 2A,D). The 3 d RT treatment also accumulated excess nutrients, with NO_3_ concentrations between 100 and 150 μM. Nitrate concentrations in the 1 d RT treatments were elevated relative to the source water, but concentrations were generally between 40 and 80 μM (Fig. 2A,D). Patterns of enrichment were similar for PO_4_ (Fig. 2C,F), while NH_4_ concentrations were drawn down relative to source water (Fig. 2B,E).

**Fig. 3.**
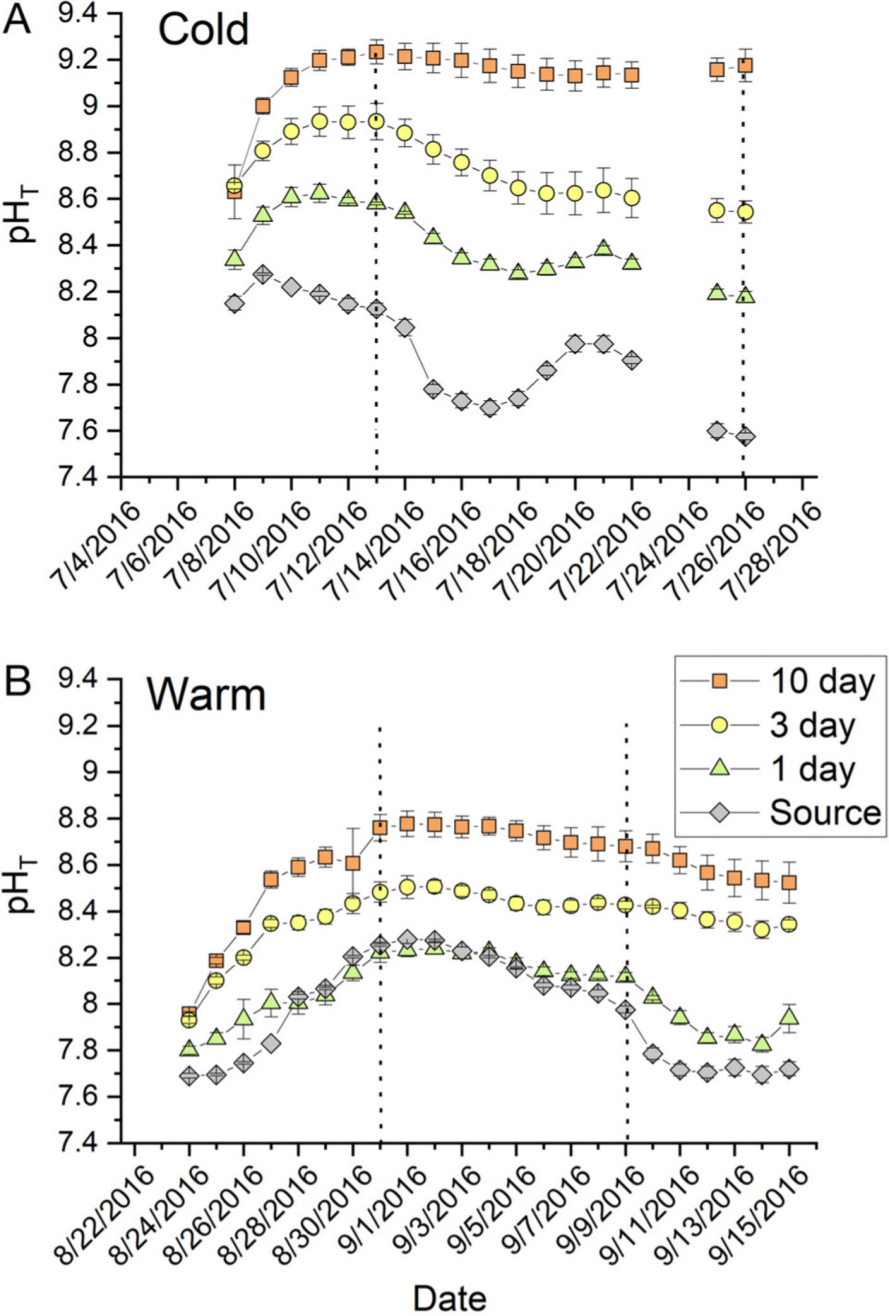
Time series (mean ± SE; n = 3) of total scale pH (pH_T_) from the (A) cold and (B) warm experiments. Values are presented for each residence time treatment as well as the source water coming into the mesocosms; the error bars may be smaller than the symbol. Dotted lines represent sampling of full carbonate chemistry. Dates are given as mo/d/yr

**Fig. 4.**
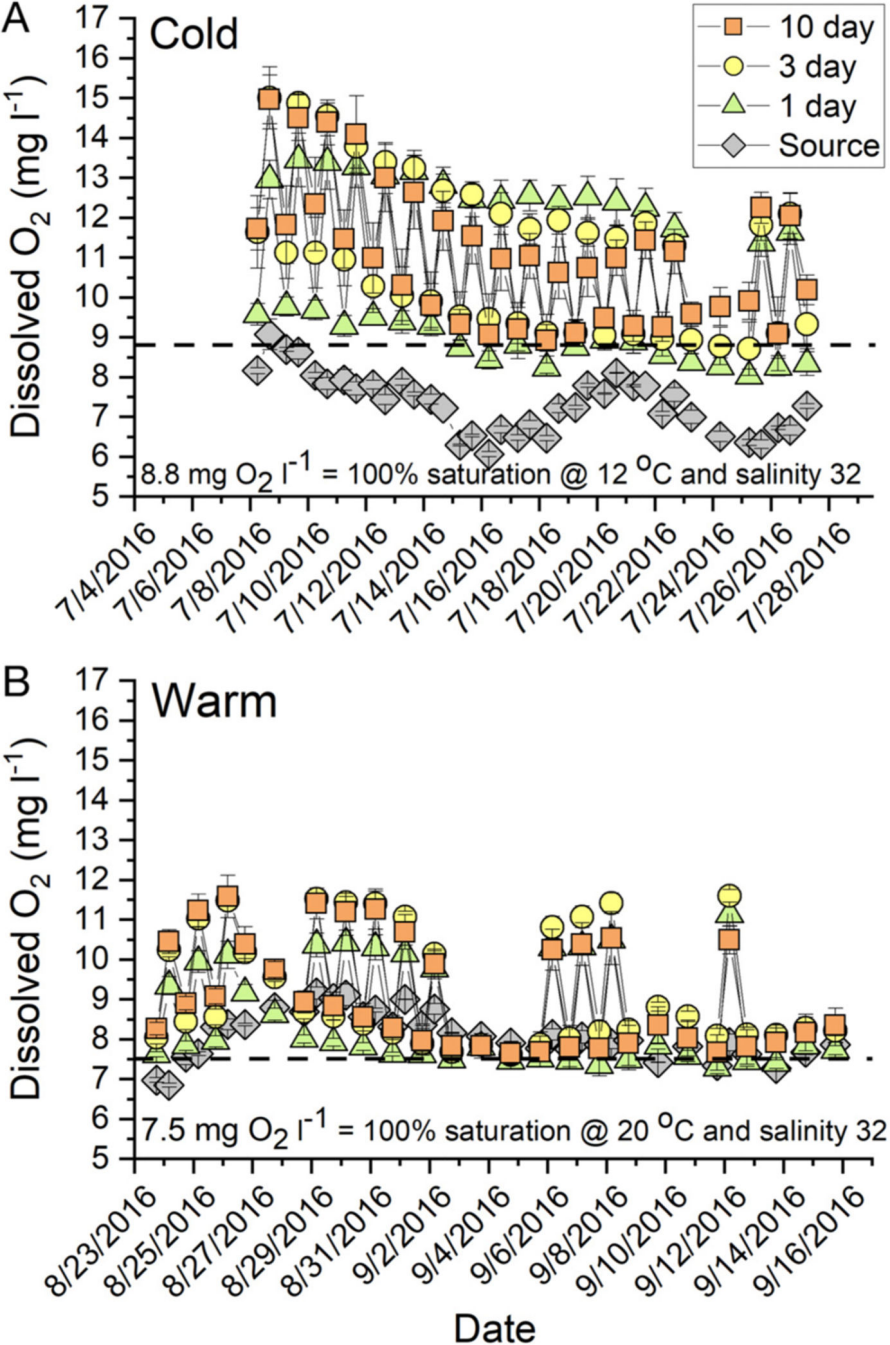
Diurnal time series of mean ± SE (n = 3) dissolved O_2_ concentrations (mg 1^−1^) during the (A) cold and (B) warm experiments. Morning values were sampled in the dark; afternoon values were sampled during the light period. Dotted line represents 100% O_2_ saturation at the experimental temperature and salinity. Dates are given as mo/d/yr

Within about 7 d of initiating the experiments, treatment tanks exhibited increased pH_T_ and DO, with photosynthesis drawing down DIC and raising pH_T_ and DO (Figs. 3 & 4). In the cold experiment, the 1 d RT tanks exhibited pH_T_ offset of about 0.5 pH_T_ units from the source water but followed similar patterns associated with a switch from relaxation to upwelling conditions as illustrated by the shift to lower source water pH_T_ (drop in pH_T_ from 8.0 to 7.7 on 15 July 2016; Fig. 3). In all treatments, diurnal pH_T_ variations on the order of 0.3 to 0.5 units (data not shown) were observed; however, these swings were small relative to treatment effects and will not be discussed further. The 3 d RT tanks were influenced by the source water pH_T_, but the dynamics were muted by photosynthetic activity causing pH_T_ to range be tween 8.5 and 8.8. In the 10 d RT treatment, the average pH_T_ reached 9.2 and remained high for the duration of the experiment (Fig. 3). Similar patterns were ob served during the warm experiment (Figs. 3 & 4), with source water tracking a wind field relaxation event from about 28 August to 10 September 2016, as reflected in the pH_T_ values ranging between about 8.0 and 8.3 (Fig. 3). The pH_T_ in the 3 d RT systems was intermediate between the 1 d and 10 d RT, with 10 d systems having pH_T_ values >8.6 (Fig. 3).

In all treatments and temperatures, mesocosm tanks quickly became supersaturated with respect to O_2_ (Fig. 4). During the light period, DO was strongly supersaturated even at 1 d RT and decreased during the dark period, but generally remained supersaturated for the duration of the experiments (Fig. 4). Conditions in the 1 d RT tanks strongly reflect the influence of the source water, as illustrated by the considerable overlap of the source water data with the 1 d RT pH_T_ and DO data. Mediated by photosynthetic activity, the 3 and 10 d RT mesocosms exhibited substantial deviations in pH_T_ and DO from the source water.

Carbonate chemistry analyses showed that the source waters feeding the mesocosms were characterized by lower pH_T_ and higher CO_2 (aq)_ than any of the treatments, an expected response if photosynthesis was influencing the carbonate chemistry in the mesocosms. On both July sampling dates, 10 d RT tanks had <1 μmol CO_2 (aq)_ kg^−1^ (Table 2), which was 5 to 15 times lower than in the 1 d RT tanks. During the warm experiment, on both sampling dates, 10 d RT had ≤2.3 μmol CO_2 (aq)_ kg^−1^ (Table 2), which was about 75% lower than the 1 d RT treatment (Table 2). Bicarbonate (HCO_3_^−^) concentrations also decreased with increasing RT, with 10 d RT tanks having concentrations 53 to 56% lower than the source water (Table 2). Calculated carbonate (CO_3_^2–^) ion availability consistently exhibited highest concentrations in the 10 d RT tanks in both experiments (Table 2). Photosynthetic production in the mesocosm tanks altered the relative availability of carbon species available to support photosynthesis. In both experiments, anomalously low alkalinity was noted in the 10 d RT tanks (Table 2). Decreased alkalinity exhibited a linear relationship with decreasing salinity because of freshwater dilution from the nutrient additions (Fig. S2).

### 3.2. Phytoplankton and surface microalgae

**Fig. 5.**
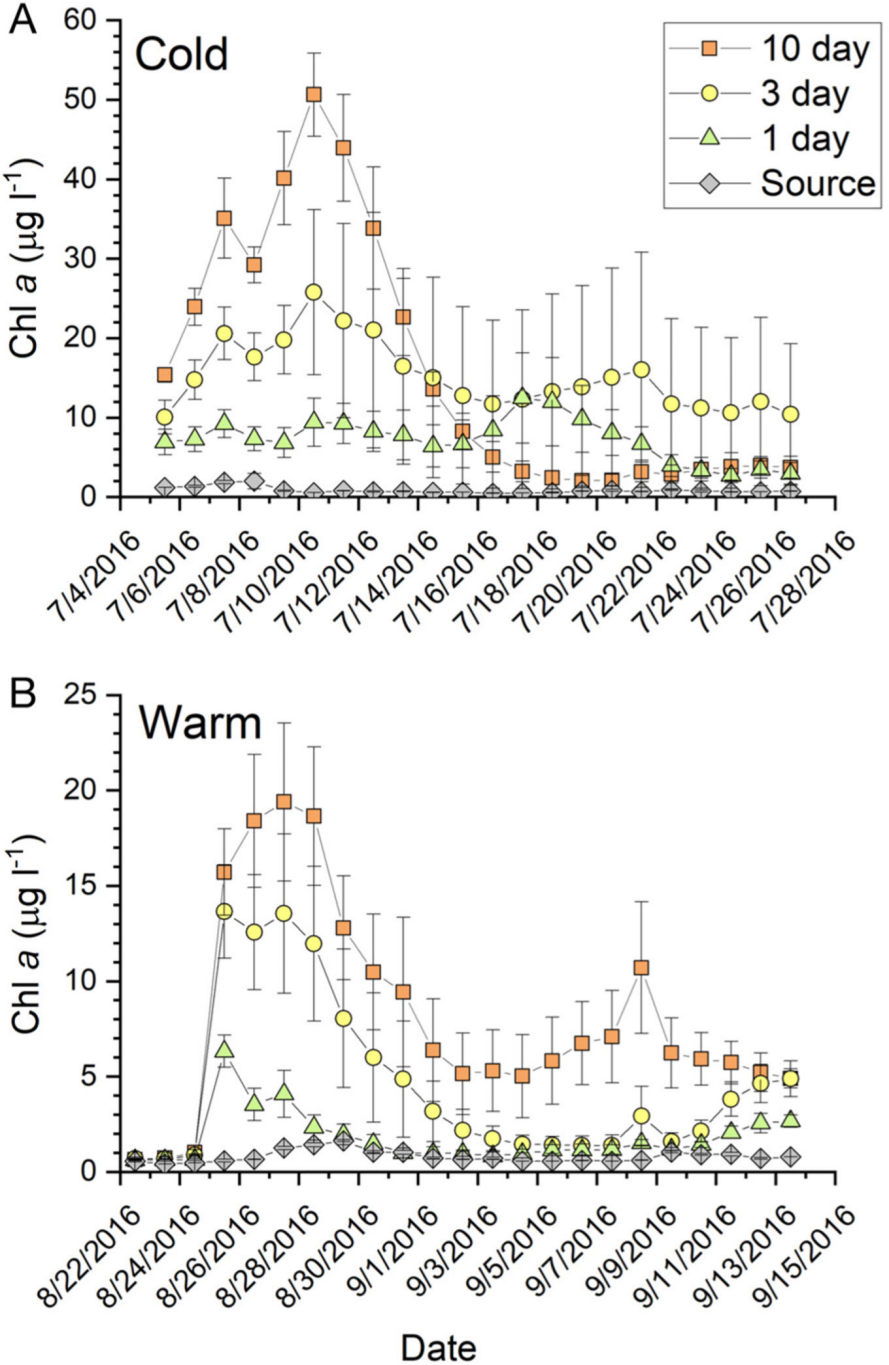
Time series (mean ± SE; n = 3) of water column chlorophyll *a* in the (A) cold and (B) warm experiments. Note that the *y*-axis scale is experiment specific. Values are presented for each residence time treatment as well as the source water coming into the mesocosms; error bars may be smaller than the symbol. Dates are given as mo/d/yr

Water column chl *a* concentration ranged between about 5 and 15 μg l^−1^ (Fig. 5) when tanks were inoculated with cultured phytoplankton. The mean daily chl *a* concentration in the source water for the mesocosms was always <1 μg l^−1^. Phytoplankton inoculum dynamics were variable. In the cold experiment at 1 d RT, the chl *a* concentration was elevated for about 2 wk, with subsequent sampling dates only slightly elevated above the source (Fig. 5). In the 3 d RT treatment, chl *a* concentration increased rapidly to a maximum of about 25 μg l^−1^ and subsequently decreased to a variable plateau (mean ~10 μg chl *a* l^−1^) but remained elevated relative to the other treatments (Fig. 5). Chl *a* concentrations in the 10 d RT tanks spiked shortly after inoculation at about 50 μg l^−1^ and decreased over the course of the experiment until they were ~2 μg l^−1^, slightly elevated relative to the source water. During the warm experiment, chl *a* dynamics were similar, although the magnitude of the bloom in the 10 d RT tanks was about 50% lower than during the cold experiment (Fig. 5). The plankton culture used to inoculate treatments was a cold water ‘weedy’ culture that may not have done well under the warmer water conditions of the experiment.

SMA exhibited strong, statistically significant responses to both temperature and RT (Table 3; see Table S2). Mean SMA in the cold experiment were about 3-fold greater than in the warm experiment (56.7 ± 7 vs. 17.8 ± 4.1 mg chl *a* tank^−1^, respectively). A Tukey post hoc test indicated that SMA at 10 d RT (24.9 ± 8.4 mg chl *a* tank^−1^) was significantly lower (p < 0.05) than at 1 d RT (50.4 ± 12 mg chl *a* tank^−1^).

### 3.3. Growth, biomass, and density

Eelgrass leaf growth, total biomass, and shoot standing stock (g DW shoot^−1^) generally exhibited strong, statistically significant responses to temperature (Table 3; Table S1). Total mean *Zostera marina* biomass in the cold tanks was about 32% larger (differing by ~8 g DW) than total biomass in the warm tanks. We also evaluated the standing stock (g DW shoot^−1^) for ‘terminal’ or adult shoots and laterals or ‘daughter’ shoots (Table S1). Lateral shoot weight was significantly greater (p < 0.05) at cold temperatures (0.041 ± 0.003 g DW shoot^−1^) compared to warm temperatures (0.016 ± 0.008 g DW shoot^−1^). Statistical analysis indicated that the Temp × RT interaction was significant for the terminal shoot weight (Table S1), but main effects were not significant, suggesting no clear pattern. Leaf growth rates ranged between 0.6 and 19.3 mg DW shoot^−1^, with plants at warm temperatures having significantly faster mean growth rates (Table 3; Table S1) of 10.1 ± 1.6 versus 6.5 ± 1.2 mg DW shoot^−1^ than plants at cold temperatures. Likewise, mean leaf elongation rates also exhibited faster growth at warm temperatures (6.9 ± 0.5 versus 4.0 ± 0.3 cm^2^ shoot^−1^ d^−1^, respectively). Additionally, new leaf tissue areal weight also exhibited a significant temperature response (Table S1), as cold temperature mean values were 2.2 ± 0.15 mg DW cm^−2^; while in the warm experiment, mean values were 1.8 ± 0.04 mg DW cm^−2^ (Table 3), about 20% lower.

**Fig. 6.**
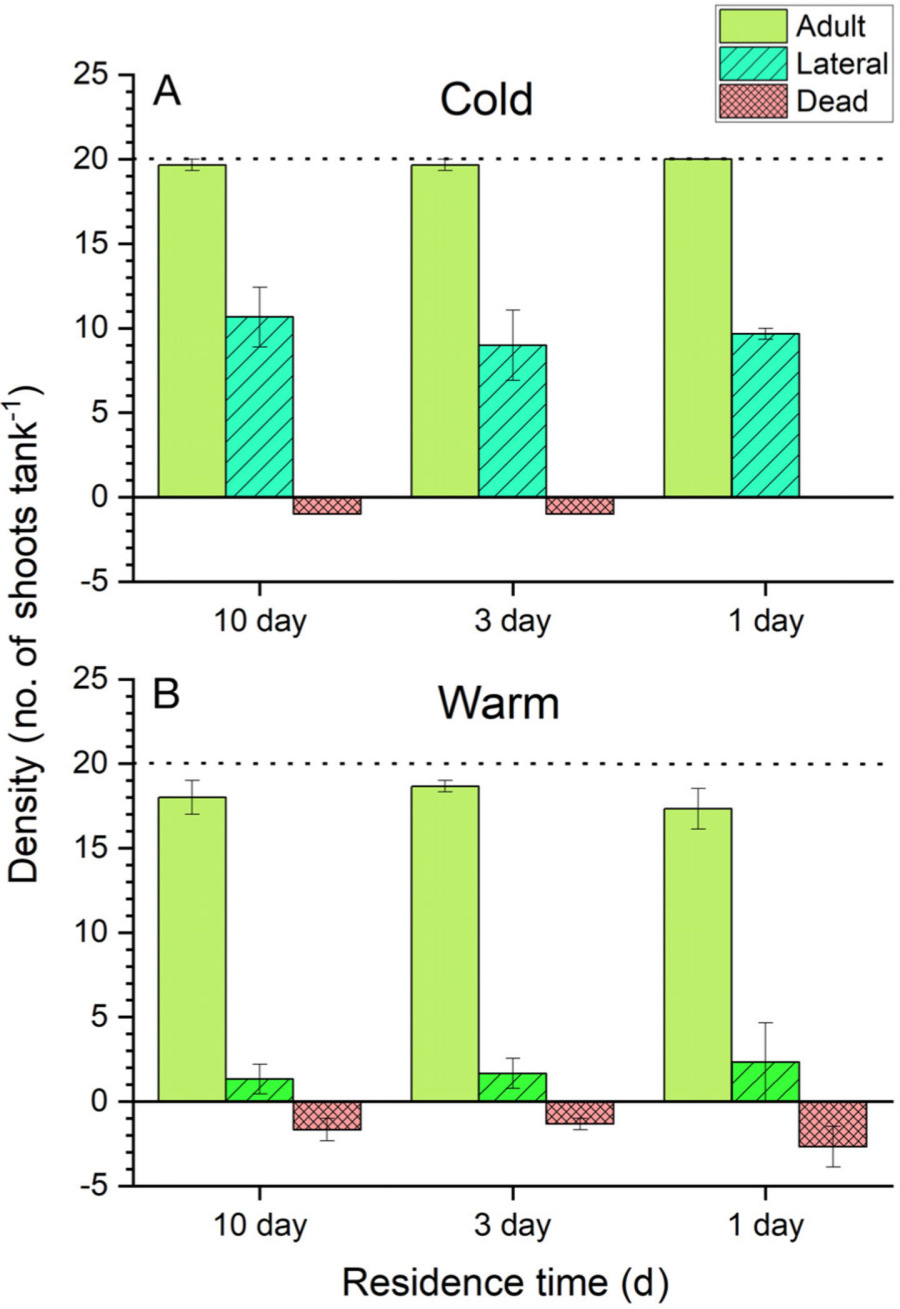
*Zostera marina* density (mean ± SE; n = 3) of adult, lateral, and dead shoots (no. tank^−1^) for the (A) cold and (B) warm experiments. Dotted line represents the initial planting density of 20 shoots per tank

We evaluated multiple density metrics, adult shoot density, lateral density, and the number of dead shoots (Fig. 6). All density metrics exhibited strong, statistically significant (p < 0.05) responses to temperature (Table S1). Each tank started with 20 *Z. marina* shoots at the beginning of the experiment. By the end of the cold and warm experiments, average shoot densities were 29.6 ± 0.8 and 19.7 ± 1.1 shoots tank^−1^, respectively (Fig. 6). Lateral shoot density (no. laterals tank^−1^) was strongly affected by temperature (Table S1). Mean lateral density at the end of the cold experiment was 9.7 ± 0.8 shoots tank^−1^, compared to 1.8 ± 0.8 lateral shoots tank^−1^ in the warm experiment. Likewise, the death of terminal shoots was strongly influenced by temperature (Table S1), with the mean number of dead shoots being 0.2 ± 0.1 tank^−1^ in the cold experiment and 1.9 ± 0.5 tank^−1^ in the warm experiment. In terms of total shoot numbers during the cold experiment, only 2 terminal shoots out of 180 died (−1%) while almost 88 laterals were formed (+48%). In contrast, in the warm experiment, 17 out of 180 shoots died (−9%) and 16 laterals were formed (+8%).

Green macroalgae biomass and growth responded to both temperature and RT treatments (Table 3; Table S2). Mean GMA biomass was about 15% greater in the warm experiment (14.6 ± 0.9 g DW ) than in the cold experiment (12.4 ± 0.8 g DW), while mean growth rates were about 30% greater in the warm vs. the cold experiment (364 ± 36 vs. 259 ± 38 mg DW d^−1^, respectively; Table 3). Tukey post hoc testing indicated that GMA biomass and growth varied with RT. After accounting for temperature, mean GMA biomass at the 1 d RT (15.2 ± 0.9 g DW) was greater (*q*-value = 4.29; p = 0.025) than at the 10 d RT (11.5 ± 0.7 g DW). Similarly, mean GMA growth rates at 1 d RT (389 ± 37 mg DW d^−1^) were greater (*q* = 4.1; p = 0.033) than at 10 d RT (226 ± 31 mg DW d^−1^).

### 3.4. Tissue chemistry

*Z. marina* leaf tissue δ^13^C and %C exhibited significant treatment effects (Table 3; Table S1). In the cold experiment, mean *Z. marina* leaf tissue was isotopically lighter (−9.2 ± 0.2‰), with lower mean carbon content (33.7 ± 0.2%) than in the warm experiment (−7.9 ± 0.1‰ and 35.3 ± 0.2% C). The Tukey post hoc test (*q* = 4.45; p = 0.006) indicated that the differences in %C between the 1 and 3 d RT were significant (34.1 ± 0.2 vs. 35.0 ± 0.2% C, respectively). Post hoc testing indicated that mean δ^13^C values were different (p < 0.05) for all 3 RTs (10 d = −8.5 ± 0.2; 3 d = −7.9 ± 0.2; 1 d = −9.4 ± 0.2‰). *Z. marina* leaf tissue δ^15^N and %N exhibited significant treatment effects (Table S1). In the cold experiment, mean *Z. marina* leaf tissue %N (2.9 ± 0.05) was significantly higher than in the warm experiment (2.7 ± 0.05%N). Mean leaf tissue δ^15^N varied between RT treatments; leaf tissue at 10 d RT was isotopically enriched (NO_3_ δ^15^N = +48‰) relative to 1 d RT (10 d: 16.1 ± 0.5 vs. 1 d: 14.4 ± 0.4‰; *q* = 3.64; p = 0.031). Leaf C:N ratio was significantly lower in the cold experiment (13.8 ± 0.2) than in the warm experiment (15.4 ± 0.3).

*Z. marina* leaf and rhizome sugar concentrations responded to changes in temperature regimes but not water RTs (Table 4; Table S1). The total sugar concentrations in leaf tissue were about 2-fold higher at 12°C (ca. 65 mg [g DW]^−1^) than at 20°C (Table 4; Table S1, p < 0.05). Leaf sucrose levels were very low, ranging between 3 and 15 mg (g DW)^−1^, accounting for 10–20% of the total leaf sugars, while fructose consistently accounted for about 50% of the total leaf sugars. Total sugar concentrations in rhizome tissue ranged between 205 and 241 mg (g DW)^−1^ (Table 4). Rhizome sugar concentrations at 20°C were 2 to 15% higher than rhizome sugar concentrations at 12°C (Table 4; Table S1, p = 0.004); however, there were no differences among RT treatments (Table S1). Rhizome sucrose concentrations ranged between 110 and 191 mg (g DW)^−1^, accounting for 50 to 80% of the total soluble sugar (Table 4) in the rhizomes.

GMA thallus tissue had δ^13^C values ranging between −14.5 and −6.5‰; statistical analysis detected a significant Temp × RT interaction, indicating that the δ^13^C becomes lighter with increasing temperature and RT. GMA thallus δ^15^N values ranged between +22.3 and +32‰ and exhibited a statistically significant temperature effect (Table 3; Table S2). Mean thallus δ^15^N was 24.7 ± 0.7‰ in the cold experiment and 29.8 ± 0.7‰ in the warm experiment (Table 3). Heavy δ^15^N values were anticipated given that the δ^15^N of the added NO_3_ was +48.14 ± 0.12‰. Thallus %C (~35%), %N (~4.5%), and C:N ratio (~9) exhibited no significant response to either temperature or RT treatments.

### 3.5. Relative contributions of seagrass and macroalgae

The relative contributions of *Z. marina* and GMA to total biomass (ratio of *Zm*:GMA) did not vary among RT treatments (Table 3), but were different between cold and warm experiments. In the cold experiment, *Z. marina* was the dominate macrophyte by about 2:1 (by biomass). In contrast, in the warm experiment, *Z. marina* was slightly dominant (*Zm*:GMA ratio was ~1.25:1; Table 3), indicating no substantial shifts in macrophyte dominance based on biomass. Although seagrass epiphytes were not quantified directly, observations indicate that eelgrass in the warm experiment had almost no visible epiphytes. In contrast, during the cold experiment, there was a thin but noticeable layer of epiphytes on the blades. This is consistent with the SMA data which exhibited significant temperature and RT effects (Table 3; Table S2).

## 4. DISCUSSION

Eelgrass eutrophication responses to nutrient loading can be more nuanced than simple light limitation, and require integration of biogeochemistry and plant physiology to elucidate response mechanisms. Contrary to our original hypothesis, algae did not become the dominant plant form, even though GMA did exhibit increased growth and biomass at shorter RT with higher CO_2 (aq)_ concentrations. We observed increased and continuous biogeochemical stresses (nutrient accumulation, extreme CO_2_ limitation, and O_2_ super-saturation) across all RT treatments, which had unexpected impacts on eelgrass. Eelgrass losses were associated with high pH_T_ and CO_2 (aq)_ concentrations of <10 μmol kg^−1^ CO_2 (aq)_, but not with high nutrients. Changes in eelgrass and algal response metrics (biomass, density, growth) were strongly associated with temperature. Eelgrass and GMA tissue chemistry (sugars, δ^13^C, δ^15^N, C:N ratio) exhibited patterns consistent with the hypothesis that carbon availability was greater at short RT. Very low *Zostera marina* leaf sugar concentrations are consistent with extreme photosynthetic CO_2 (aq)_ limitation. We suggest that the effect of extremely low environmental carbon concentrations and increased respiration from warm temperatures and other physiological processes can lead to internal carbon limitation and shoot mortality. Consequently, in some cases, CO_2 (aq)_ limitation may control eutrophication expression and is discussed further in Section 4.1.

### 4.1. Extreme carbon limitation vs. nitrate toxicity

In a pioneering mesocosm eutrophication study, Burkholder et al. (1992) described what they hypothesized to be a direct toxic impact of low level NO_3_ enrichment (<10 μM NO_3_) to *Z. marina* plants. They hypothesized that plant death was due to an internal carbon imbalance caused by the inability to down-regulate nitrate reductase which utilizes energy (NADPH) to convert NO_3_ to NH_4_ and C-skeletons during synthesis into amino acids (Touchette & Burkholder 2000b), effectively leading to carbon starvation (Burkholder et al. 2007). If the inability to down-regulate nitrogen uptake drives eelgrass into negative carbon balance (‘nitrate toxicity’) by plant metabolic processes at <10 μM NO_3_, then *Z. marina* should not have survived in this experiment or other extreme nutrient manipulation experiments (Kaldy 2014). Further, we would not expect eelgrass to be present in Pacific Northwest estuaries of the USA, where nitrate concentrations regularly exceed 30 μM during upwelling-favorable conditions (Brown & Ozretich 2009, Lee & Brown 2009). Seagrass mapping work indicates that outside of a few areas, general seagrass distribution within many Oregon estuaries has been stable since the 1970s (Cortright et al. 1987, Kaldy 2014). Similarly, eelgrass distribution in Puget Sound has been fairly stable as well (Shelton et al. 2017, Christiaen et al. 2019).

An alternative explanation for eelgrass loss in these experimental systems was extreme photosynthetic CO_2 (aq)_ limitation leading to negative carbon balance, particularly at warm temperatures. Although the total DIC pool is large (~2.2 mmol), the amount of inorganic carbon accessible for eelgrass as CO_2 (aq)_ is not capable of supporting maximum rates of photosynthesis (Beer & Koch 1996, Invers et al. 2001, Palacios & Zimmerman 2007, Mvungi et al. 2012). Zimmerman et al. (1997) showed that *Z. marina* is CO_2 (aq)_-limited in seawater at 14 μmol kg^−1^ CO_2 (aq)_, while McPherson et al. (2015) showed that *Z. marina* photosynthesis is carbon-limited at about 1 mmol kg^−1^ HCO_3_^−^. In both experiments presented here, CO_2 (aq)_ concentrations in experimental tanks ranged from 1 to 13.5 μmol kg^−1^ CO_2 (aq)_, with most values <8.5 μmol kg^−1^ CO_2 (aq)_. HCO_3_^−^ values were on the order of 800 to 1100 μmol kg^−1^ (Table 2), and measured pH_T_ values were between 8 and 9.2 (Fig. 3). Consequently, eelgrass photosynthesis in our experiments were likely strongly CO_2 (aq)_-limited. For comparison, Fig. 1 in Burkholder et al. (1992) shows that pH ranged between 8.2 and 9.2 in North Carolina mesocosm tanks. As a result, eelgrass photosynthesis in the Burkholder experiment was also likely carbon-limited, which may have confounded their interpretation of plant death.

Seagrass photophysiology has been a topic of intense research (Hemminga & Duarte 2000, Larkum et al. 2006), and recently there has been a focus on carbon acquisition (Larkum et al. 2017, Zimmerman 2017). Many seagrasses, including *Z. marina*, have a limited capacity to use extracellular carbonic anhydrase to catalyze the disassociation of H_2_CO_3_ to CO_2 (aq)_ and H_2_O (Beer & Koch 1996), but this mechanism cannot sustain light-saturated photosynthesis (Arnold et al. 2017). Further, increased temperature de creases the ratio of photosynthesis to respiration (P:R), leading to reduced net primary production (Lee et al. 2007, Zimmerman 2017). Under high O_2_ conditions, photo respiration (oxygenation of RUBISCO) becomes more prevalent, thereby de creasing net photosynthetic carbon fixation (Buchanan et al. 2000, Buapet et al. 2013). Photorespiration also reduces eelgrass photosynthetic quantum efficiency or light-harvesting capability, further reducing photo synthetic carbon fixation (Celebi-Ergin et al. 2021). All experimental tanks were continuously supersaturated with respect to DO, with concentrations between 8 and 15 mg O_2_ l^−1^ (Fig. 4). Consequently, it is likely that photorespiration also influenced eelgrass internal carbon balance. Combined CO_2_-limited photosynthesis, reduced P:R ratio from warming, and likely photorespiration probably pushed the eelgrass plants into negative carbon balance, especially at 20°C.

Plant tissue sugar concentrations are a sensitive indicator of internal carbon limitation. *Z. marina* leaf and rhizome tissues in both experiments exhibited depleted total sugar concentrations compared to historical data collected from the same field site (Table 4), especially leaf tissue in plants grown at 20°C. Total sugar concentrations in seagrass leaf and rhizome tissue are on average about 100 to 275 mg (g DW)^−1^, and sucrose generally accounts for about 90% of the total sugar pool (Touchette & Burkholder 2000a). Historical data from August 2001 (Table 4) were very similar to the generic data summarized by Touchette & Burkholder (2000a). Measured *Z. marina* leaf total sugar concentrations were 30 to 60% lower than the generic seagrass average (Touchette & Burkholder 2000a) and are similar to values from heat-stressed plants (31–66 mg [g DW]^−1^) observed in late summer in Chesapeake Bay (Burke et al. 1996). Additionally, sucrose accounted for 10–20% of total leaf sugar content, while fructose accounted for >50% of the total leaf sugars. Sugar content in historical samples exhibited equal quantities of fructose and glucose with sucrose accounting for 80–90% of the total (Table 4). For comparison, Cabello-Pasini et al. (2002) found that leaf sucrose was ~228 mg (g DW)^−1^ (as- suming 6.7 g fresh weight [g DW]^−1^) after 14 d of growth in darkness. The very low leaf sugar concentrations, especially at 20°C (Table 4) and fructose dominance indicate that sucrose was not being transported from the stored reserves, suggesting internal carbon limitation. Measured rhizome total sugar concentrations were about 15–24% lower than either the generic average (Touchette & Burkholder 2000a) or historical samples from Yaquina Bay. Sucrose in the rhizome accounted for 10–40% less of the total sugar pool than expected, further supporting the internal carbon limitation hypothesis. We hypothesize that under severe CO_2 (aq)_ limitation, recent fixed photosynthate was insufficient to support metabolism, and the plants may have been catabolizing starch, leading to higher than expected concentrations of glucose and fructose. Unfortunately, we were not able to measure carbohydrate content of initial plants or the starch concentrations in experimental plants.

We also expected that changes in the δ^13^C signature of the plants would provide an indication of CO_2 (aq)_ limitation. Eelgrass δ^13^C signatures typically range between about −6 and −12‰ (Hemminga & Mateo 1996), with local values around −11.77 ± 0.58‰ (mean ± SE, n = 14, J. Kaldy unpubl. data), while typical local values for GMA are −15.63 ± 0.59‰ (n = 12, J. Kaldy unpubl. data). We expected that as carbon limitation increased (concomitant pH_T_ increase), the plant tissue δ^13^C would become isotopically heavier (more positive). The *Z. marina* tissue δ^13^C data exhibited patterns consistent with this hypothesis; at 20°C, plants were about 1.3‰ heavier and experienced higher CO_2 (aq)_ concentrations than during the 12°C experiment (Tables 2 & 3). Additionally, there was a statistically significant ~1‰ de crease in leaf δ^13^C values with increased RT (Table 3; Table S1). The isotopically lighter values are a result of more isotopic discrimination at the higher CO_2 (aq)_ concentrations (Table 2). The heavier δ^13^C signature was also evident in the algal data and was clearest in the cold experiment, where there was a 5.8‰ difference between the 10 d and the 1 d RT. The heavy algae δ^13^C may indicate increased use of HCO_3_^−^ as a carbon source. Although the δ^13^C DIC pool is often considered fixed, the stable isotope ratio is ion specific in the carbonate system, and HCO_3_^−^ is about 8‰ heavier than the δ^13^C CO_2_ (Zeebe & Wolf-Gladrow 2001). Since *Z. marina* prefers CO_2 (aq)_, the ~1‰ shift in the eelgrass leaf δ^13^C is likely not related to HCO_3_^−^ utilization and coupled with the very low sugar concentrations provides 2 independent lines of evidence that internal carbon limitation was the likely cause of eelgrass decline.

### 4.2. Demographic implications of mortality and lateral growth patterns

Documenting and understanding seagrass demographics has been an active area of research (Hemminga & Duarte 2000) often complicated by the interplay of external forcing and intrinsic plant characteristics (Mascaró et al. 2014). However, few studies have determined the drivers of the observed changes in demographic parameters (although see Marbà & Duarte 2010, Mascaró et al. 2014). Colarusso (2006) showed that increased lateral shoot formation in *Z. marina* transplants was related to initial rhizome carbohydrate reserves, with larger reserves leading to more laterals. Differences in lateral shoot production among our experimental treatments has important implications for eelgrass demography. We hypothesize that *Z. marina* plants in the cold experiment were able to increase shoot density through efficient carbon recycling and lower respiratory demands at cooler temperatures. At 12°C, almost all of the adult eelgrass shoots survived, and 88 new laterals were formed (Fig. 6). In contrast, under warm temperatures and intense carbohydrate limitation (Table 4), the eelgrass population barely remained stable and had a net decline in shoot number (17 adult shoots died, and 16 laterals were generated). Interestingly, Burkholder et al. (1992) observed a similar pattern in a fall experiment, with a positive change in shoot densities as temperatures decreased below 20°C (see Figs. 5 & 8 in Burkholder et al. 1992). Increased respiration at warm temperatures likely puts the adult shoots in a more severe negative carbon balance, ultimately leading to mortality (8.5% decrease in adult shoots). Eriander (2017) and Palacios & Zimmerman (2007) also observed that light limitation reduced carbohydrate storage and resulted in reduced lateral branching. Consequently, chronic sublethal stress impacts not only the individual shoot physiology, but also the demographic characteristics of the eelgrass population which could select for a robust and resilient population.

### 4.3. Community consequences

Alterations in community composition resulting in algal dominance are among the most obvious responses to excessive nutrient loading (McGlathery 2001). In our experiments, temperature had a large impact on the plant community structure. Likewise, RT played an important role in GMA growth even though it did not become the dominant plant component, with larger biomass and faster growth at short RTs (Table 3; Table S2). This pattern was opposite of our original hypothesis but is consistent with our carbonate chemistry data, since the highest algal biomass and growth occurred at the highest CO_2 (aq)_ concentrations in the short RTs (Table 2). Warm temperatures favored algal biomass development and growth, while 12°C had more robust eelgrass biomass and higher SMA biomass (Table 3). McGlathery et al. (2007) suggested that marine plant communities alter biogeochemical processes that influence system level nutrient retention.

Carbonate chemistry, especially extreme CO_2 (aq)_ limitation is another pathway through which macrophyte communities can be impacted by nutrient-driven eutrophication. Algae often possess CCMs that allow them to utilize both CO_2 (aq)_ and bicarbonate (Raven et al. 2011), providing a competitive advantage and would be expected to dominate these systems. GMA thallus growth rates were about an order of magnitude higher than *Z. marina* leaf growth rates (Table 3), which is consistent with the ability of GMA to utilize bicarbonate more efficiently than eelgrass. However, unlike many other experiments, *Z. marina* remained the dominant macrophyte by biomass (*Zm*:GMA, Table 3) suggesting that GMA biomass accumulation was not keeping up with growth rates, even though grazers were absent. We hypothesize that either longer experiments would yield different results or that some other factor was limiting algal production, or possibly that production of secondary compounds (e.g. dopamine, DMSP; Van Alstyne et al. 2014, Van Alstyne 2018) may have been an algal energy sink. Short-term studies provide critical insight to short-term physiological responses; however, extended experiments are likely to produce important responses not observed at short time scales (Zimmerman 2021). The results presented here provide valuable hypotheses which can inform future experimental designs for longer-term experiments.

Another potential impact of severe internal carbon limitation may have been the structural integrity of the plant biomass. Reductions in eelgrass leaf areal mass (mg DW cm^−2^) associated with warm temperatures were observed in these experiments. *Z. marina* growth rates were similar to those reported from previous mesocosm experiments (7–12 mg DW shoot^−1^ d^−1^), but leaf areal mass (1.76–2.17 mg DW cm^−2^; Table 3) was generally less than previously reported for both temperatures (2–2.5 mg DW cm^−2^; Kaldy et al. 2017). Understanding the combined impacts of carbon limitation, thermal stress, and nitrogen loading at the plant level is important for the development and utilization of indicators for detecting impacts to macrophyte communities.

Although eelgrass has a limited capacity to utilize HCO_3_^−^ (Larkum et al. 2017), GMA are widely accepted to have CCMs that can sustain photosynthesis at high pH conditions (Raven et al. 2011). Additionally, GMA generally also have lower minimum light requirements (Sand-Jensen 1988, Valiela et al. 1997) and a higher affinity for nutrients (Duarte 1995, Valiela et al. 1997). These traits may convey a competitive advantage to GMA and provide a plausible physiological basis for their dominance under eutrophic conditions. Under persistent conditions, this could lead to an alternate stable state favoring algal producers. One caveat of this work is that these were short-term experiments (21 d) that showed a potential shift in resource allocation to favor formation of new shoots during cooler temperatures and toward respiration and maintenance of existing shoots under warmer temperatures. Thus, thermal regime may also impact plant responses, with potential consequences for population demographics. Longer-term experiments are logistically challenging and may be expected to reveal more deleterious impacts on seagrass at both temperature regimes due to carbon limitation.

## Supplementary Material

SI

## Figures and Tables

**Table 1.** Average daily ambient nutrient loading (μmol d^−1^) based on concentration × flow rate; treatment manipulated loads were added daily as described in Section 2. Residence times were 10, 3, and 1 d. Note: source water values represent nutrient concentrations (μM), not loads. SE based on n = 24

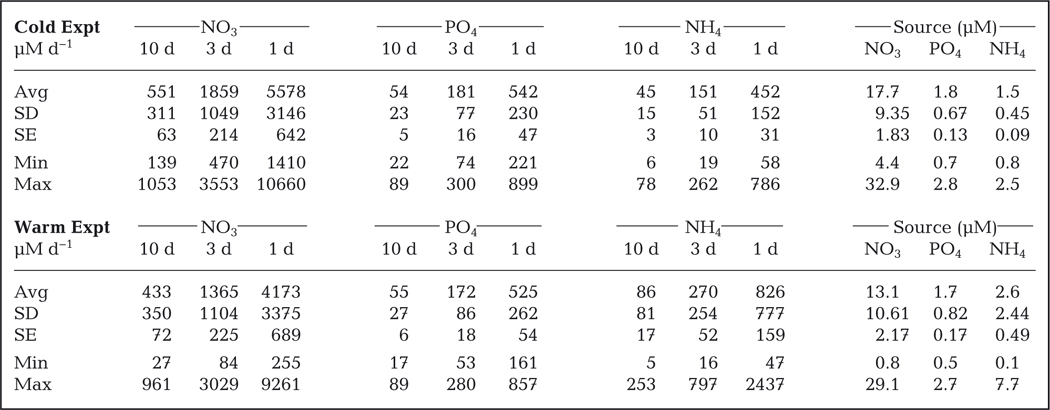

**Table 2.** Summary of complete carbonate system (mean ± SE) calculated from discrete samples analyzed for *p*CO_2_ and TCO_2_ from selected sampling dates during the 2 mesocosm experiments. The cold experiment was conducted during July; the warm experiment was conducted in August/September of 2016 (dates are given as mo/d/yr). Total scale pH (pH_T_) values were truncated, not rounded. Measured values in **bold**

Date	Residence time (d)	Alkalinity (μeq kg^−1^)	TCO_2_ (μmol kg^−1^)	CO_2 (aq)_ (μmol kg^−1^)	*p*CO_2_ (μATM)	Bicarbonate (μmol kg^−1^)	CO_3_ (μmol kg^−1^)	pH_T_	Sample size (n)

7/13/2016	10	2001.8 ± 34.7	**1311.1 ± 21.3**	0.93 ± 0.04	**22.2 ± 0.9**	856.5 ± 14.1	453.7 ± 13.3	8.94 ± 0.01	6
	3	2148.1 ± 5.7	**1540 ± 17.4**	1.86 ± 0.18	**44.6 ± 4.3**	1130.2 ± 33	408 ± 15.9	8.77 ± 0.03	6
	1	2187 ± 3.7	**1785.4 ± 9.6**	4.9 ± 0.2	**118.7 ± 5.8**	1508.6 ± 16.7	272 ± 7.6	8.46 ± 0.02	6
	Source	2226.5 ± 27.0	**1992.9 ± 25.6**	12.3 ± 0.2	**327.1 ± 6.2**	1815 ± 23.8	165.6 ± 2.4	8.11 ± 0.01	4
7/26/2016	10	1877.6 ± 12.7	**1229.9 ± 23.9**	0.88 ± 0.09	**20.9 ± 2.2**	806.4 ± 32.0	422.6 ± 9.2	8.95 ± 0.03	4
	3	2170.3 ± 2.4	**1760.7 ± 8.9**	4.63 ± 0.20	**110.5 ± 4.6**	1479.1 ± 14.8	276.9 ± 6.2	8.48 ± 0.01	6
	1	2217.6 ± 3.8	**2002.8 ± 12.5**	13.5 ± 1.1	**320.8 ± 33.2**	1837.4 ± 21.4	151.9 ± 10.1	8.12 ± 0.03	6
	Source	2257 ± 3.0	**2226.7 ± 0.9**	49.9 ± 1.3	**1194.8 ± 33.2**	2123.1 ± 0.8	53.7 ± 1.3	7.60 ± 0.01	3
8/31/2016	10	2089.4 ± 7.7	**1455.3 ± 26.3**	1.69 ± 0.18	**52.8 ± 5.6**	1031 ± 38.8	422.6 ± 13	8.68 ± 0.03	6
	3	2202.6 ± 11.8	**1674.4 ± 21.1**	3.23 ± 0.25	**99.8 ± 7.2**	1312.4 ± 31.2	358.8 ± 12	8.50 ± 0.02	6
	1	2206.8 ± 8.8	**1837.2 ± 15.2**	6.62 ± 0.47	**205.4 ± 14.4**	1576.5 ± 24.9	254.1 ± 11.2	8.27 ± 0.03	6
	Source	2264.1 ± 15.5	**1973.2 ± 15.7**	9.26 ± 0.16	**232.5 ± 4.1**	1762 ± 15.2	202 ± 1.3	8.24 ± 0.00	4
9/9/2016	10	2057.6 ± 16.5	**1502.6 ± 33.1**	2.27 ± 0.22	**70.4 ± 7**	1128.1 ± 42.8	372.2 ± 10.2	8.60 ± 0.03	6
	3	2175.4 ± 12.6	**1716.8 ± 20.6**	4.16 ± 0.24	**128 ± 8.2**	1400.4 ± 25.4	312.2 ± 5.4	8.42 ± 0.02	6
	1	2233.5 ± 4.5	**1915.1 ± 4**	8.46 ± 0.18	**259.4 ± 5.5**	1684.8 ± 6.1	221.9 ± 3.3	8.19 ± 0.01	6
	Source	2240.4 ± 6.3	**2097.5 ± 4**	21.2 ± 0.25	**529.9 ± 6.4**	1966.5 ± 2.8	109.8 ± 1.6	7.93 ± 0.01	3

**Table 3.** Biomass, growth rates, stable isotope ratios, and C:N ratios for macrophyte and microalgal biomass for the warm and cold experiments. All values are mean ± SE. DW: dry weight; GMA: green macro algae; SMA: surface microalgae. Note differences in units

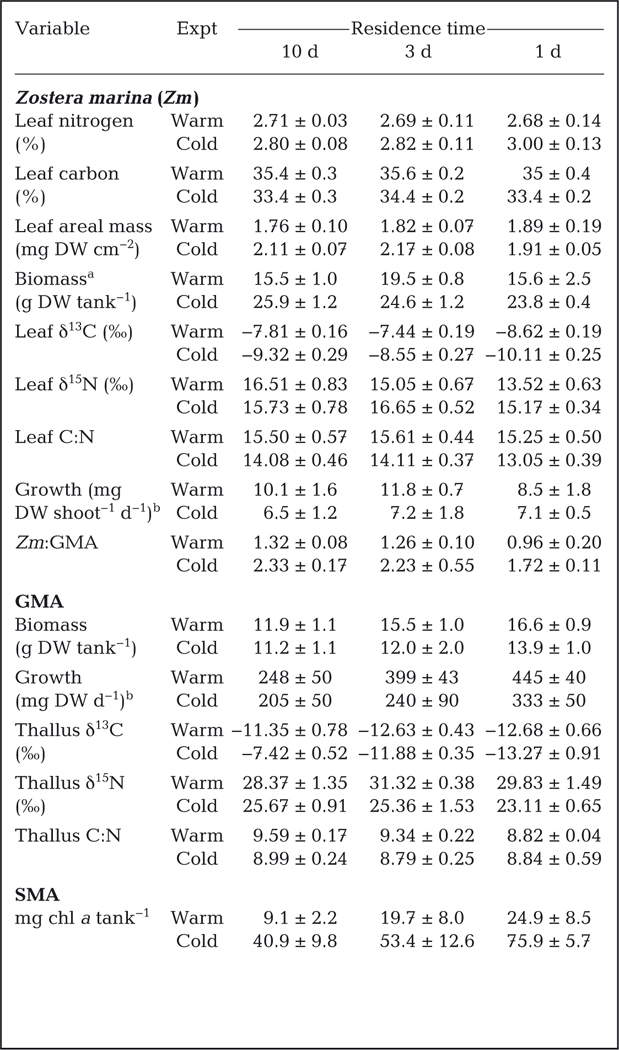

a Total biomass of above- and below-ground tissues combined

b SE based on n = 3

**Table 4.** Mean ± SE (n = 9) sugar concentrations (mg per g dry weight) in composite samples of *Zostera marina* tissues measured by HPLC. Field plants were collected in August 2001 and analyzed using similar HPLC protocols (n = 16); their sugar concentrations and % contribution to total sugars are presented for comparison (J. Kaldy unpubl. data)