# Supplementary material for: Carbon limitation in response to nutrient loading in an eelgrass mesocosm: influence of water residence time
Source: Mar Ecol Prog Ser. Author manuscript; Available in PMC 2023 May 12. (PMC9347230; doi:10.3354/meps14061)
Supplement: SI [file NIHMS1808632-supplement-SI.pdf]

Table S1. Summary of 2 way ANOVA results for *Z. marina* metrics. Main effects were temperature (Temp; warm and cold), water residence time (RT; slow, medium, fast), and the interaction term Temp x RT for each dependent variable. Df = degrees of freedom, MS = mean square. **Bold** font for p-values < 0.05. [a] Above + below-ground tissues

Tissue	Dependent	Source	df	MS	*F*-ratio	p
Total	Biomass [a]	Temp	1	280.0	52.93	**<0.0001**
	(gdw)	RT	2	8.1	1.53	0.2551
		T x RT	2	10.6	2.00	0.1774
		Error	12	5.2		
		Total	17			
Leaf	Terminal shoot weight	Temp	1	0.005	2.57	0.134
	(gdw shoot^{-1})	RT	2	0.004	1.92	0.187
		T x RT	2	0.014	6.58	**0.011**
		Error	12	0.002		
		Total	17			
	Lateral shoot weight	Temp	1	0.002	7.79	**0.016**
	(gdw shoot^{-1})	RT	2	0.0003	1.01	0.393
		T x RT	2	0.0003	1.14	0.352
		Error	12	0.0003		
		Total	17			
	Total density	Temp	1	440.0	39.60	**0.0003**
	(shoots tank^{-1})	RT	2	0.055	0.005	0.995
		T x RT	2	2.05	0.185	0.833
		Error	12	11.11		
		Total	17			
	Lateral density	Temp	1	288	39.57	**0.0004**
	(shoot tank^{-1})	RT	2	0.888	0.122	0.886
		T x RT	2	2	0.274	0.764
		Error	12	7.277		
		Total	17			
	Dead shoots	Temp	1	12.5	11.25	**0.005**
	(shoots tank^{-1})	RT	2	0.388	0.35	0.711
		T x RT	2	1.166	1.05	0.379
		Error	12	1.111		
		Total	17			
	Tot. sugars	Temp	1	16389	66.6	**<0.0001**
	(mg gdw^{-1})	RT	2	58.6	0.23	0.788
		T x RT	2	28.8	0.12	0.889
		Error	48	245.8		

	Total	53			
Sucrose ($mg\ gdw^{-1}$)	Temp	1	693	20.8	**<0.0001**
	RT	2	60.8	1.8	0.171
	T x RT	2	61.5	1.8	0.168
	Error	48	33.2		
	Total	53			
Glucose ($mg\ gdw^{-1}$)	Temp	1	1310	53.4	**<0.0001**
	RT	2	15.1	0.618	0.542
	T x RT	2	5.4	0.221	0.802
	Error	48	24.5		
	Total	53			
Fructose ($mg\ gdw^{-1}$)	Temp	1	4289	79.1	**<0.0001**
	RT	2	30.4	0.562	0.573
	T x RT	2	53.0	0.979	0.382
	Error	48	54.1		
	Total	53			
Elongation ($cm\ shoot^{-1}\ d^{-1}$)	Temp	1	166.6	22.15	**0.0001**
	RT	2	3.8	0.50	0.602
	T x RT	2	12.9	1.71	0.186
	Error	81	7.5		
	Total	86			
Growth ($mg\ dw\ shoot^{-1}\ d^{-1}$)	Temp	1	236.5	10.40	**0.001**
	RT	2	18.11	0.796	0.454
	T x RT	2	13.56	0.596	0.553
	Error	81	22.74		
	Total	86			
Areal weight ($mg\ dw\ cm^{-2}$)	Temp	1	3.00	5.22	**0.024**
	RT	2	0.422	0.73	0.483
	T x RT	2	1.14	1.98	0.144
	Error	81	0.57		
	Total	86			
$\delta^{13}C$ (‰)	Temp	1	37.82	49.27	**<0.0001**
	RT	2	13.86	18.07	**<0.0001**
	T x RT	2	0.58	0.75	0.472
	Error	79	0.76		
	Total	84			
%C (%)	Temp	1	54.42	50.74	**<0.0001**
	RT	2	4.65	4.34	**0.016**

		T x RT	2	1.25	1.17	0.314
		Error	79	1.07		
		Total	84			
	$\delta^{15}N$ (‰)	Temp	1	17.86	2.91	0.091
		RT	2	26.38	4.30	**0.016**
		T x RT	2	15.86	2.58	0.081
		Error	79	6.12		
		Total	84			
	%N (%)	Temp	1	0.699	6.52	**0.012**
		RT	2	0.099	0.93	0.397
		T x RT	2	0.094	0.88	0.417
		Error	79	0.107		
		Total	84			
	C:N	Temp	1	57.34	19.14	**<0.0001**
		RT	2	4.84	1.61	0.204
		T x RT	2	1.54	0.515	0.599
		Error	79	2.99		
		Total	84			
Rhizome	Tot. sugar (mg gdw^{-1})	Temp	1	7215	9.10	**0.004**
		RT	2	131.7	0.16	0.847
		T x RT	2	1129	1.42	0.250
		Error	48	792		
		Total	53			
	Sucrose (mg gdw^{-1})	Temp	1	49668	86.8	**<0.0001**
		RT	2	721	1.2	0.292
		T x RT	2	1361	2.4	0.103
		Error	48	571		
		Total	53			
	Glucose (mg gdw^{-1})	Temp	1	3005	49.7	**<0.0001**
		RT	2	21.6	0.36	0.700
		T x RT	2	15.6	0.25	0.772
		Error	48	60.3		
		Total	53			
	Fructose (mg gdw^{-1})	Temp	1	6905	81.3	**<0.0001**
		RT	2	152.7	1.8	0.176
		T x RT	2	41.3	0.5	0.617
		Error	48	84.8		
		Total	53			

Table S2. Summary of ANOVA results for green macroalgae (GMA) and surface microalgal (SMA) metrics. Main effects were temperature (Temp; warm and cold), water residence time (RT; slow, medium, fast), and the interaction term Temp x RT for each dependent variable. Df = degrees of freedom, MS = mean square. **Bold** font used to denote P value < 0.05.

Plant	Depend	Source	Df	MS	F-ratio	P value
GMA	Biomass	Temp	1	23.75	5.21	**0.041**
	(gdw)	RT	2	21.26	4.66	**0.031**
		T x RT	2	3.40	0.74	0.494
		Error	12	4.55		
		Total	17			
	Growth	Temp	1	0.049	5.23	**0.041**
	(mg dw d^{-1})	RT	2	0.040	4.23	**0.040**
		T x RT	2	0.005	0.54	0.592
		Error	12	0.009		
		Total	17			
	$\delta\ ^{13}C$	Temp	1	8.36	6.8	**0.022**
	(‰)	RT	2	21.5	17.5	**0.0002**
		T x RT	2	8.0	6.5	**0.011**
		Error	12	1.2		
		Total	17			
	$\delta\ ^{15}N$	Temp	1	118.0	30.3	**0.0001**
	(‰)	RT	2	5.5	1.4	0.279
		T x RT	2	6.8	1.7	0.214
		Error	12	3.8		
		Total	17			
SMA						
	SMA	Temp	1	6802	31.6	**<0.0001**
	(mg Chl *a* $tank^{-1}$)	RT	2	974	4.5	**0.034**
		T x RT	2	168	0.78	0.477
		Error	12	214		
		Total	17			

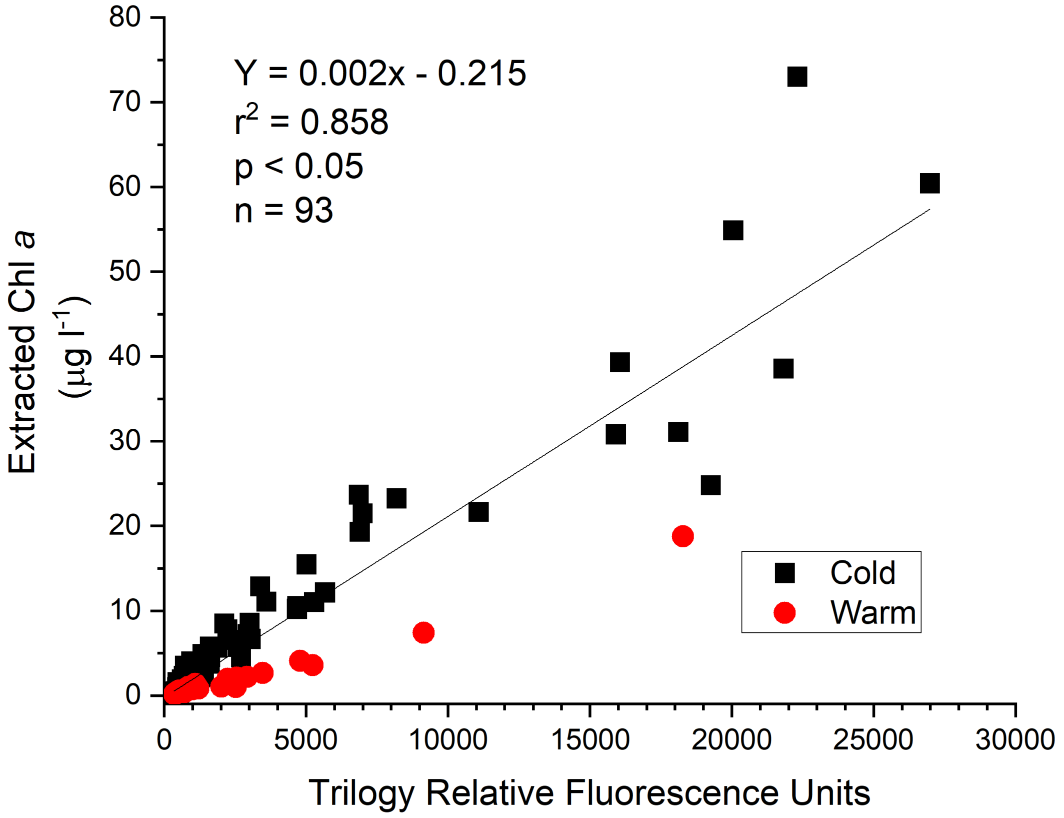

Figure S1. Relationship between Relative Fluorescence Units (RFU) and extracted Chl *a* concentration from the same sample during the warm and cold mesocosm experiments. Combined data were used to convert measured RFU into estimated Chl *a* concentrations.

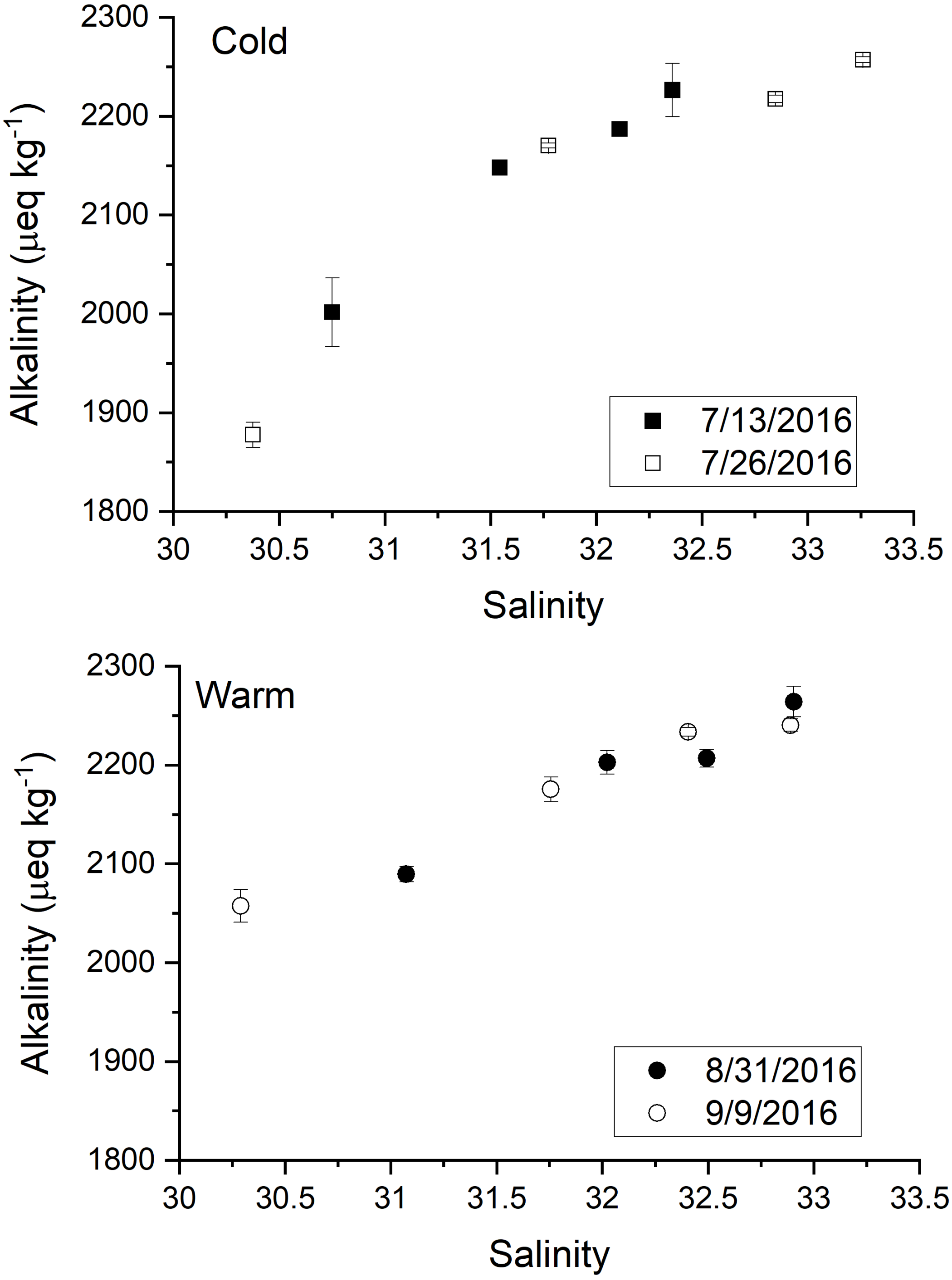

Figure S2. Plot of alkalinity (μeq kg^{-1}) versus salinity for the 10 d residence time treatments during the warm and cold experiments. Lowered salinity was an artifact of daily addition of 4 liter nutrient solution aliquots in Milli-Q water.
